# Tobacco use among Kyrgyzstan medical students: an 11-year follow-up cross-sectional study

**DOI:** 10.1186/s12889-017-4547-6

**Published:** 2017-07-04

**Authors:** Nurlan Brimkulov, Denis Vinnikov, Zhamilia Dzhilkiadarova, Aigerim Aralbaeva

**Affiliations:** 1 Kyrgyz State Medical Academy after I.K. Akhunbaev, Akhunbaev street 92, Bishkek, 720020 Kyrgyz Republic; 2 Al-Farabi Kazakh National University, Al-Farabi avenue 71, Almaty, 050040 Kazakhstan; 3 Public Association “Lung Health”, Akhunbaev street 92, Bishkek, 720020 Kyrgyz Republic

**Keywords:** Smoking, Waterpipe, Medical students, Training, Tobacco control

## Abstract

**Background:**

Medical students are the first line active force to combat tobacco epidemic, but they may suffer from high smoking prevalence and wrong attitude themselves. The aim of the study was to assess the effect of current curriculum on smoking behavior of medical students in Kyrgyzstan.

**Methods:**

20% random sample of all 6 years of the School of Medicine in Kyrgyz State Medical Academy were interviewed in spring 2016. The questionnaire included sections on tobacco products consumption and knowledge and attitude to counseling. We verified smoking status with exhaled CO measurement using Bedfont Smokelyzer.

**Results:**

In 618 students (48% female), the overall daily cigarette smoking prevalence was 21% (34% in males and 6% in females), being highest in years 1 and 3 and least in year 5 (prevalence difference 14%). With very low smokeless products and electronic cigarettes use prevalence, ever-smoking prevalence of waterpipe use was very high, reaching 85% in 6-year male students with alarmingly high prevalence in female students also. Only 74% students responded there was 100% evidence of harmful effects of tobacco, unchanged throughout the course of study.

**Conclusions:**

The use of tobacco products, especially smoking waterpipe, in Kyrgyzstan medical students remains very high. Coupled with poor knowledge and high demand for more information, this demonstrates urgent need for more active and advanced training on tobacco control in medical school.

## Background

Providing support and professional counseling are essential components of successful Framework Convention on Tobacco Control (FCTC) implementation, overall smoking prevalence reduction and achieving long-term goals in reducing the burden of smoking-related deaths and morbidity [1]. The role of medical professionals has been discussed widely and was considered no less important than the population-level interventions. High smoking prevalence among medicals in low-income countries, however, may impede proper interaction between a smoker willing to quit and a medical professional, where model behavior may play crucial role. Despite induction of advanced tobacco dependence treatment curricula in some institutions around the world, medical students may still exhibit high smoking prevalence and lack appropriate counseling skills. Our cross-sectional study of Kyrgyzstan medical students 11 years ago [2] revealed a worrisome trend in smoking prevalence increase with advanced years of study and low preparedness for counseling. At that time, daily smoking prevalence ranged from 9% at year 1 to 40% at year 6. Moreover, those students demonstrated relatively poor knowledge of the evidence of the association of smoking with disease. Thus, only 64% of current smokers agreed there existed 100% evidence of the association of smoking with morbidity.

Since then, new modules in medical student’s curriculum in Kyrgyz State Medical Academy were introduced and covered some of the tobacco dependence treatment options. Thus, four hours on tobacco control were introduced in the curriculum for year 5 students, as well as 18-h elective course on tobacco control was offered. Moreover, new clinical protocol on tobacco dependence treatment was discussed in the Department of Evidence-Based Medicine in the Ministry of Health and proposed with further approval. Considering local medications availability, this protocol recommended the use of cytisine as an add-on to structured counseling program, and the efficacy of this medication was confirmed in the local randomized controlled trial [3]. However, widespread use and marketing of waterpipe (hookah) for smoking and electronic cigarettes undermined effective tobacco restriction and ban activities on the national level, because the national law on tobacco control did not consider those. Aggressive marketing of waterpipe smoking in Kyrgyz Republic is mainly tailored at the most susceptible groups, such as students [4], including future medical professionals, because of high profits that the owners of small waterpipe cafes make. Unlike waterpipe, smokeless tobacco may cost much less to a user, nevertheless, its prevalence remains uncertain, and due to social pressure of being a role model medical student, students may use this form as an alternative to openly demonstrated conventional cigarette smoking.

Therefore, we planned this study with the aim to assess the effect of current curriculum on smoking behavior of medical students in Kyrgyzstan.

## Methods

### Design and subjects

This study was constructed as a follow-up of the previously published study [2[using similar methodology in both sample selection and the questionnaires. For detailed description of the venue and research approach as well as the baseline smoking prevalence, a paper from 11 years ago may provide more information [2]. This time we enrolled 618 students of the medical school (20% random sample of the registrar list of current students), who were randomly selected from the students’ list. Of note, internal medicine syllabus for 5-year students included a brief course of lectures and discussions tobacco dependence verification, evaluation and treatment, including basic information on tobacco control as a public health issue. The study was approved by the Ethical Committee of Kyrgyz State Medical Academy.

We collected material via self-administered questionnaires in February, March and April 2016. Selected students from all six years of basic medical course were interviewed, and then were asked to exhale in a portable carbon monoxide (CO) monitor to verify their smoking status. All paperwork and measurements were done after classes, and all questionnaire were distributed in hardcopies.

### Questionnaire

The questionnaire was offered to all selected students in Russian and consisted of 29 questions divided into two parts. Part A was a compilation of questions on smoking status, including smoking cigarettes, waterpipe smoking and the use of smokeless tobacco, as well as Fagerstrom Test for Nicotine Dependence (FTND) combined with two questions on motivation to quit and previous attempts to do so. Cigarette smoking status was defined as follows: cigarette never-smokers were subjects who never tried any cigarette in their lifetime. Cigarette ever-smokers, on opposite, were those who smoked at least one cigarette in their lifetime, and may have either continued to smoke till the time of the interview, or quit. Daily cigarette smokers were those smoking at least one cigarette a day at a time of the interview. Waterpipe smoking status was defined using similar approach: waterpipe ever-smokers were the ones who tried waterpipe at least once in their lifetime. Those who smoked waterpipe at least once daily were considered daily waterpipe smokers. Those smoking waterpipe less than once a day but at least once a week were weekly waterpipe smokers. Finally, students smoking waterpipe less than once a week but at least once a month were monthly waterpipe smokers. Similar classification relates to electronic cigarette users, stratifying them to ever-, and daily electronic cigarette users. Smokeless tobacco users were classified to ever- (used at least once in their lifetime) and never-users.

Part B was a group of 13 questions on knowledge and attitude. We asked whether waterpipe smoke was more harmful than conventional smoked tobacco; constituents of smokeless tobacco; whether, to their knowledge, smoking was proved to be associated with cancer, chronic obstructive pulmonary disease, ischemic heart disease and other chronic conditions. We also asked if a medical professional should advise all smoking patients cease smoking, and if such an advice could motivate a smoker quit and if medical students needed more training of counselling and smoking cessation pharmacotherapy. One question was on the choice of a class medical students would prefer to have smoking cessation methods covered. In this part B, we also asked if medical students knew what FCTC was. Finally, one more question was on alcohol consumption.

### Exhaled CO measurement

We measured exhaled CO in all students who responded to the questionnaire with Bedfont piCO Smokelyzer (Bedfont, UK). We required an interval at least 15 min after the last smoked cigarette in daily smokers, whereas in all the rest we measured exhaled CO immediately after the questionnaire. As mandated by the manual, after deep inhalation, a subject holds his breath for 15 s and then exhales into the device, where data from electrochemical sensor are displayed on a sensor. CO readings were used to confirm smoking status of cigarette and waterpipe users. Our accepted cut-off level for daily cigarette or waterpipe smokers in this study was 9 ppm; therefore, all those with CO 9 ppm and more were considered daily cigarette or waterpipe smokers (as reported by the subject). Students with exhaled CO less than 9 ppm were classified into the categories of either occasional, or never-smokers as of their self-reported status.

### Statistical analysis

All answers from the questionnaire were coded as either binomial or continuous variables. We compared groups within the variables stratifying them into subgroups of sex, year of study, answers (yes/no) using Mann-Whitney U test or t-test in cases when data were distributed normally. Alternatively, we tested the role of chance in continuous variables using Wilcoxon test. For a multiple year of study comparisons, we used main-effects ANOVA and reported probability of chance when comparing one variable across all six years of study with one *p*-value. Statistically significant difference was present when ANOVA *p*-value test was below 0.05. All tests were performed in NCSS 10 (Utah, USA).

## Results

### Smoking prevalence and behavior

**Fig. 1:**
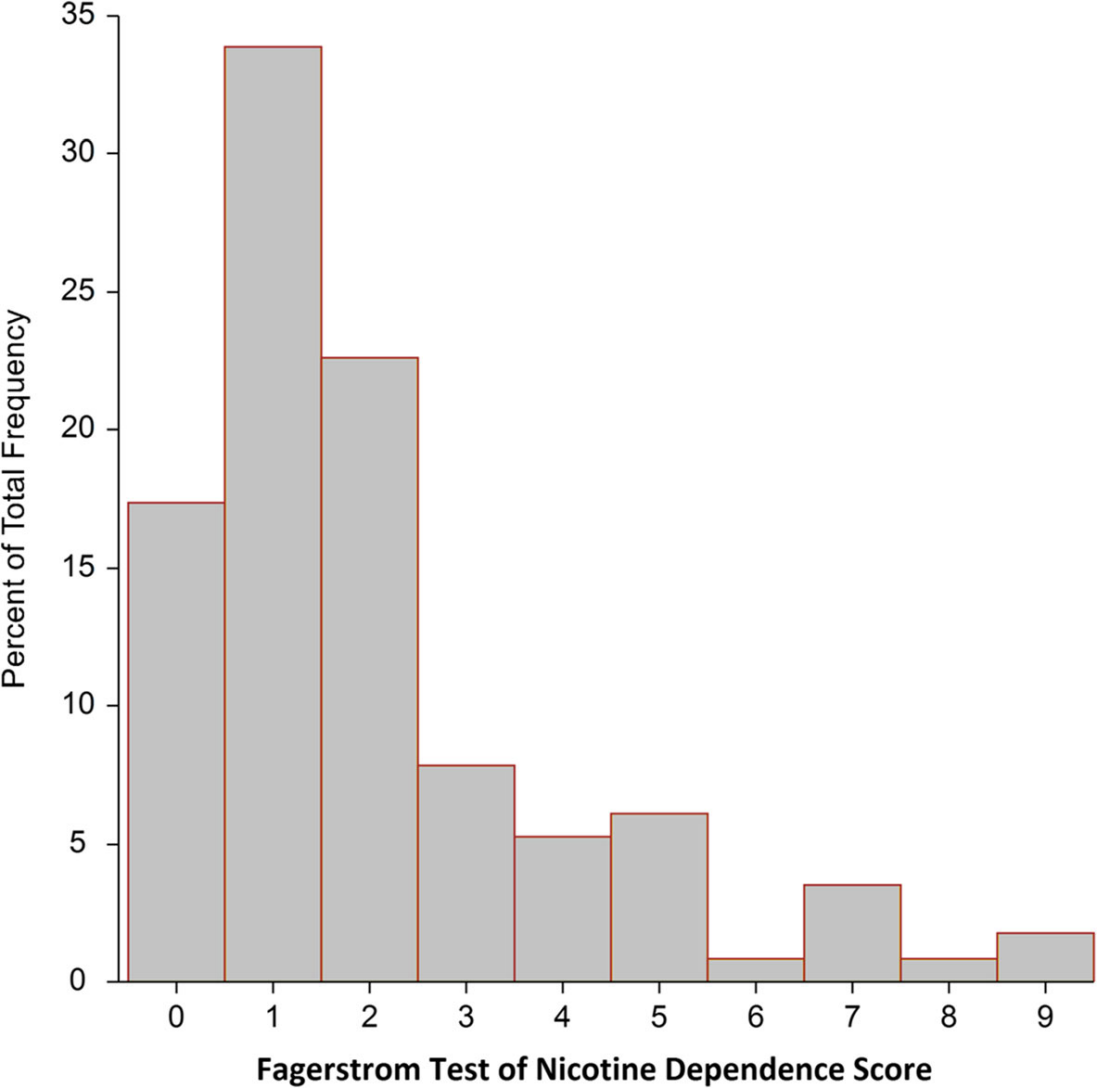
Histogram of FTND score in the full cohort of medical students

Of 618 enrolled students, 48% were females (*N* = 322). The majority of Kyrgyzstan medical students ever tried at least one tobacco product. With the mean daily cigarette smoking prevalence 21%, female medical students showed 4-fold lower daily cigarette smoking prevalence compared to their male counterparts, with this trend persisting throughout the entire course of study. Almost every other medical student was an ever-cigarette smoker, with prevalence ranging from 32 to 50% (the mean ever-smoking cigarette prevalence 43%) (Table 1). In the current study, we found U-shape both daily and ever-smoking cigarette prevalence with the least number of cigarette smokers on the fifth year of study. With the overall daily cigarette smoking prevalence almost remaining unchanged, we failed to see growing daily smoking prevalence with advanced year of study, observed before. Cigarette daily smokers had in general very low smoking dependence with a median FTND score of 1 and the mean 2.1 ± 2.0 (left-screwed), and FTND score 3 being 75th percentile (Fig. 1).

**Table 1:** The prevalence of use of tobacco products in medical students

	Overall	Year 1	Year 2	Year 3	Year 4	Year 5	Year 6
N	618	103	102	104	104	101	104
Females, N (%)*	296 (48)	42 (40)	41 (40)	51 (50)	54 (52)	58 (57)	50 (48)
Cigarettes							
Cigarette never-smokers, N (%)*	351 (57)	56 (54)	58 (56)	51 (50)	62 (60)	69 (68)	55 (53)
Cigarette ever-smokers, N (%)*	267 (43)	48 (46)	45 (44)	51 (50)	42 (40)	32 (32)	49 (47)
*Cigarette ever-smokers in male students, N (%)*	209 (65)	42 (68)	38 (61)	36 (71)	33 (66)	25 (58)	35 (65)
Cigarette ever-smokers in female students, N (%)*	58 (20)	6 (14)	7 (17)	15 (29)	9 (17)	7 (12)	14 (28)
Cigarette daily smokers, N (%)*	128 (21)	28 (27)	22 (21)	28 (27)	17 (16)	13 (13)	20 (19)
*Cigarette daily smokers in male students, N (%)*	109 (34)	23 (37)	21 (34)	21 (41)	16 (32)	10 (23)	18 (33)
Cigarette daily smokers in female students, N (%)*	19 (6)	5 (12)	1 (2)	7 (14)	1 (2)	3 (5)	2 (4)
Waterpipe							
Waterpipe ever-smokers, N (%)*	376 (61)	61 (59)	50 (49)	70 (69)	61 (59)	64 (63)	70 (67)
Waterpipe ever-smokers in male students, N (%)*	238 (74)	45 (73)	37 (60)	43 (84)	34 (68)	33 (77)	46 (85)
Waterpipe ever-smokers in female students, N (%)*	138 (47)	16 (38)	13 (32)	27 (53)	27 (50)	31 (53)	24 (48)
Waterpipe daily smokers, N (%)	14 (2)	3 (3)	6 (6)	2 (2)	2 (2)	0 (0)	1 (1)
Waterpipe weekly smokers, N (%)	27 (4)	8 (8)	0 (0)	4 (4)	5 (5)	7 (7)	3 (3)
Waterpipe monthly smokers, N (%)	98 (16)	14 (13)	18 (17)	20 (20)	13 (13)	15 (15)	18 (17)
Smokeless tobacco							
Smokeless tobacco ever-users, N (%)	37 (6)	5 (5)	9 (9)	9 (9)	9 (9)	0 (0)	5 (5)
Electronic cigarettes							
Electronic cigarette ever-smokers, N (%)*	42 (7)	13 (13)	12 (12)	6 (6)	4 (4)	3 (3)	4 (4)
Electronic cigarette daily smokers, N (%)*	12 (2)	6 (6)	4 (4)	0 (0)	2 (2)	0 (0)	No data

Note: * - *p* < 0.05 for ANOVA test comparisons across years of study

Albeit only 4% of medical students smoked waterpipe weekly, the number of waterpipe ever-smokers was alarmingly high and reached 69% in the third-year students and being 61% on average. Of note, up to 85% (6-year students) of male medical students ever smoked waterpipe, but only 0–6% of students of both sexes smoked waterpipe daily. Moreover, in female students, waterpipe ever-smoking prevalence was much higher than that of cigarettes, being 47% on average, but spiking to 53% at years 3 and 5. These rates of waterpipe ever-smoking were considerably higher than cigarette ever-smoking prevalence in the similar years of study. Even the number of students who tried waterpipe only once was still very high, and by the year 6 reached 46%. Compared to waterpipe smoking, electronic cigarettes were far less popular and only 2% of medical students used electronic nicotine delivery systems daily. Smokeless tobacco in the studied sample was the least used product, and despite some 6% use in the past, almost no students reported its regular use at a time of this survey.

### Knowledge and attitude

As 11 years ago, knowledge of smoking-related morbidity remained unacceptable. Only 74% of students were sure about unambiguous scientific evidence of a number of chronic conditions and cancer to be associated with smoking. Despite higher proportion of medical students choosing this option, we did not identify any trend of growing prevalence with advanced year of study, which should have been implied as a training outcome (Table 2). Number of students in favor of “insufficient evidence” considerably increased from an earlier study. With quite high number of students supporting medical advice to quit in clinical practice, peaking at year 5, some 23% of graduates had an alternative view. 68% medical students said they would need more information on the causative association of smoking with specific diseases, but only 63% were in need of more training on treatment, which was a notable drop compared to the preceding survey. Students were also asked what class they would prefer to have an advanced training in diseases causation related to smoking. Of those 420 who needed more, 55 (13%) would rather have that in pathologic physiology class, 146 (35%) in internal diseases class, 126 (30%) in narcology and psychiatry class, and finally 141 (34%) in non-existing “Tobacco or Health” class. FCTC and apparently related issues, such as the role of medical professionals in helping smokers quit should be better covered in the syllabus of associated classes, because only 27% of medical students had enough information on that.

**Table 2:** Knowledge and attitude of medical students

	Overall	Year 1	Year 2	Year 3	Year 4	Year 5	Year 6
N	618	103	102	104	104	101	104
Smoking causes cancer and other diseases							
100% evidence, N (%)*	459 (74)	86 (83)	75 (73)	68 (67)	73 (70)	79 (78)	78 (75)
Insufficient evidence, N (%)*	121 (20)	16 (15)	20 (19)	14 (14)	30 (29)	21 (21)	20 (19)
*No evidence, N (%)*	18 (3)	2 (2)	2 (2)	6 (6)	1 (1)	1 (1)	6 (6)
A medical should advise all patients quit smoking*	522 (84)	84 (81)	89 (86)	82 (80)	91 (88)	96 (95)	80 (77)
Such advice may be effective*	380 (61)	68 (65)	56 (54)	58 (57)	73 (70)	67 (66)	58 (56)
I need more info on smoking as a cause of disease	420 (68)	67 (64)	72 (70)	70 (69)	75 (72)	75 (74)	61 (59)
I need more info on smoking dependence treatment*	391 (63)	53 (51)	62 (60)	74 (73)	68 (65)	69 (68)	65 (63)
I am ready to take part in anti-tobacco activities	374 (61)	48 (46)	52 (50)	53 (52)	80 (77)	75 (74)	66 (63)
I know enough about FCTC*	166 (27)	10 (10)	14 (14)	14 (14)	28 (27)	80 (79)	20 (19)
Alcohol consumption							
*Never or very seldom*	128 (21)	19 (18)	20 (19)	23 (23)	19 (18)	19 (19)	28 (27)
*Sometimes*	87 (14)	9 (9)	18 (17)	19 (19)	15 (14)	10 (10)	16 (15)
*Quite often*	17 (3)	3 (3)	4 (4)	2 (2)	4 (4)	2 (2)	2 (2)

Note: * - *p* < 0.05 for ANOVA test comparisons across years of study

## Discussion

This is a follow-up of previously published study of Kyrgyz State Medical Academy medical students, in which we asked about self-reported smoking prevalence, waterpipe, smokeless tobacco and electronic cigarette use, verified rates with exhaled CO and also asked about the attitudes and knowledge of medical students. We identified new and emerging threat of dramatically high prevalence of wastepipe use. This product may even be a more popular tobacco product in medical students, whereas overall conventional smoking prevalence remained almost unchanged during the last 11 years.

Quite high weekly and monthly waterpipe smoking prevalence in this study (19%) is a novel finding, and may be indicative of two serious gaps in the training system and legislation. When the acting tobacco control law was discussed and later adopted in 2006, waterpipe smoking was probably not so prevalent, and the issue of its control was not properly addressed at that time. High prevalence in this study should foster wider discussion on the possible ways to combat growing epidemic of its use, starting with urgent amendments to the acting law to stop widespread advertisement and affordability. Because waterpipe use is strongly associated with cancer, respiratory disease and other health effects [5, 6], equal ban of waterpipe smoking should be immediately introduced in the local legislation in order to save lives now and promote healthier behavior in those who should initiate that, i.e. medical students. The evidence on the adverse effects of waterpipe smoking is sufficient, although underestimated [7], hence, more efforts are needed to translate this knowledge into mass campaigns and a clearer action plan.

Cigarette smoking in Kyrgyz students was comparable to that in many samples constructed from medical students around the globe, such as Turkish [8], German [9] students, healthcare professionals in Armenia [10], but lower when compared to a few neighboring countries of Asian region [11]. Waterpipe smoking prevalence studies differ in their estimates of current waterpipe smoking in various countries and population groups. Thus, a systematic review from 2011 showed that current waterpipe smoking prevalence was high in both school and university students, reaching 33% in Pakistan, remaining at a level of 6–8% in the United Kingdom [12], and the latter did not increase dramatically from 2008 [13]. In selected countries, nargile smoking prevalence was outstandingly high, such as in Jordan, where up to 53% of female students smoked [14]. In this survey, as compared to the study from eleven years ago, we did not demonstrate increasing daily smoking prevalence with advanced year of study, which may be indicative of some effect of recently introduced tobacco control syllabus at year 5 to cover basic tobacco control, identification and treatment of tobacco dependence. However, even despite that training, some 25% of sixth year medical students still considered poor or no evidence of harmful effects of tobacco. This leaves substantial potential for more intense intervention and reassessment of curriculum to close the gap of poor education. Such advanced training may be effective, since more than 60% of students on overall stated they needed more information on either smoking as a cause of disease or ways to treat dependence. The highest level of knowledge on FCTC and the statement that an advice should be given to every smoker to quit was recorded in 5-year students.

This study has a number of strengths. Unlike studies collecting data only from questionnaires, this study not only compared self-reported smoking prevalence, but also verified it with a biochemical marker, such as exhaled CO. Secondly, we used similar methodology and slightly amended questionnaire, as well as randomization and selection methods, which enabled unbiased comparison with the study undertaken 11 years ago. Besides, the use of a simple study design and its low cost are other essential strengths of this study, because this survey was performed in conditions of low resources, but with very high demand for reliable data to guide future interventions. Of note, very poor resources availability is a meaningful obstacle to conduct studies like this in low-income countries like Kyrgyzstan, especially cohort observations or intervention trials.

The limitations of this study originate from the study design. Because this is a cross-sectional survey, we could not ascertain the incidence of tobacco products use, therefore, we could not conclude at which year medical students increased cigarettes or waterpipe smoking intensity. We only reported prevalence of use, in order to guide prevention strategies and address most calling gaps in medical students training system. This type of study is still valuable to make comparisons with the similar design previous study, as we did. In the current study, we only compared daily and ever-smoking, which cannot fully reflect on current smoking, and given such limitation, smoking prevalence in this study should be compared to other studies only with a similar terminology. We only covered School of Medicine and could not engage students enrolled in the School of Dentistry and the School of Sanitation. Finally, our questionnaire was only available to students in Russian, which may have created additional barriers in understanding for those students, who were raised in purely Kyrgyz language environment, despite acceptable knowledge of Russian in all students a priori.

## Conclusions

In conclusion, in this follow-up study we managed to ascertain prevalence of smoking and other tobacco products use, where smoking prevalence remained almost unchanged, but a new, almost uncontrolled, threat to public health in medical students was elucidated. Waterpipe smoke is a fast growing concern in the population of Kyrgyzstan medical students and should be addressed immediately. Current curriculum, although containing elements of tobacco control and tobacco dependence identification and treatment, is insufficient and calls for intervention that is more active.

## Abbreviations

CO: carbon monoxide
FCTC: Framework Convention on Tobacco Control
FTND: Fagerstrom Test of Nicotine Dependence
